# 

**Published:** 1894-07-07

**Authors:** 


### CONTENTS.

**Vaccination: A New Research** - 287
**Around the Hospitals** - 288
**On Modern Progress in Ophthalmic Medicine and Surgery.** By Robert Brudenell Carter, F.R.C.S. - 289
**Studies in Applied Sociology.—XI.** How Workers Live—The Woollen Industry - 290
**Some Aspects of Medical Science** - 291
**Modern Medico-Psychology and Psychiatry.—**By T. S. Clouston, M.D.—IX. Forms and Classification of Mental Disease - 292
**The History and Capabilities of Herbal Simples.** By W. T. Fernie, M.D.—The Common Ragwort - 292
**Annotations.—**A Check on Public Poisoning—That Vile Indigestion—Commercial Medicine: A Prophecy Fulfilled - 293
**Notes and News** - 294
**Medical Progress and Hospital Clinics.—**Diseases of the Ear, 295; Gastric Ulcer and Its Treatment, 296; The Treatment of Wounds - 297
Medical Progress.—Kidney Diseases - 298
Progress in Surgery.—Renal Surgery - 300
General Surgery - 301
New Appliances and Things Medical - 302
**The Institutional Workshop:**
Hospital Finance.—IX. Finance Administration - 303
The Story of the Insane from Year to Year - 304
Practical Departments—Hospitals in India - 305
Hospital Festivals and Meetings—Construction Notes - 306
**Scraps and Gleanings** - 306
**Continental Notes from a Holiday Correspondent.** Paris (*continued*) - 307

EXTRA SUPPLEMENT.—THE NURSING MIRROR.

**News from the Nursing World.** — The Registered Nurses' Society—An Outside Examiner—Invalid Children—Living Pictures — Our Needlework Competition — Prize Giving to the Women Students—Old, Helpless, and Poor—Nottingham—Nurses at Bradford Infirmary—A Good Nurse Appreciated—Good Workers at Burton—Thorough - cxxxi

**On General Nursing.—**XIX. Peritonitis (*continued*) - cxxxiii

**The Mechanism of Movement.—VII.** - cxxxiv

**A Case for Assistance—Presentation** - cxxxiv

**The Royal Visit to the British Home for Incurables** cxxxv

**Notes from Victoria.** A Cheering Outlook - cxxxvi

**Nursing in Germany** - cxxxvi

**Private Nursing in Connection with a Hospital** - cxxxvii

**Notes and Queries** - cxxxvii

**Two Hours Off Duty—Notes from Holland** - cxxxviii

**Everybody's Opinion** - cxxxix

**"The Hospital" Convalescent Fund** - cxl

**For Reading to the Sick** - cxl

